# KCNQ Channels in the Mesolimbic Reward Circuit Regulate Nociception in Chronic Pain in Mice

**DOI:** 10.1007/s12264-021-00668-x

**Published:** 2021-04-26

**Authors:** Hao-Ran Wang, Su-Wan Hu, Song Zhang, Yu Song, Xiao-Yi Wang, Lei Wang, Yang-Yang Li, Yu-Mei Yu, He Liu, Di Liu, Hai-Lei Ding, Jun-Li Cao

**Affiliations:** 1 Jiangsu Province Key Laboratory of Anesthesiology, Xuzhou Medical University, Xuzhou, 221004 China; 2 Jiangsu Province Key Laboratory of Anesthesia and Analgesia Application Technology, Xuzhou Medical University, Xuzhou, 221004 China; 3 Department of Anesthesiology, The First Affiliated Hospital of Nanjing Medical University (Jiangsu Province Hospital), Nanjing, 210029 China; 4 Department of Anesthesiology, Renji Hospital, Shanghai Jiaotong University School of Medicine, Shanghai, 200000 China; 5 Department of Anesthesiology, The Affiliated Hospital of Xuzhou Medical University, Xuzhou, 221002 China

**Keywords:** Nociception, Mesocorticolimbic system, Ventral tegmental area, Brain-derived neurotrophic factor, KCNQ, Retigabine, Chronic neuropathic pain

## Abstract

Mesocorticolimbic dopaminergic (DA) neurons have been implicated in regulating nociception in chronic pain, yet the mechanisms are barely understood. Here, we found that chronic constructive injury (CCI) in mice increased the firing activity and decreased the KCNQ channel-mediated M-currents in ventral tegmental area (VTA) DA neurons projecting to the nucleus accumbens (NAc). Chemogenetic inhibition of the VTA-to-NAc DA neurons alleviated CCI-induced thermal nociception. Opposite changes in the firing activity and M-currents were recorded in VTA DA neurons projecting to the medial prefrontal cortex (mPFC) but did not affect nociception. In addition, intra-VTA injection of retigabine, a KCNQ opener, while reversing the changes of the VTA-to-NAc DA neurons, alleviated CCI-induced nociception, and this was abolished by injecting exogenous BDNF into the NAc. Taken together, these findings highlight a vital role of KCNQ channel-mediated modulation of mesolimbic DA activity in regulating thermal nociception in the chronic pain state.

## Introduction

Chronic pain has become an enormous health care issue, affecting >15% of the world population [1, 2]. The literature suggests that the mesolimbic reward system plays a critical role in regulating chronic pain [3–6]. The ventral tegmental area (VTA), a core part of the mesolimbic reward system, contains predominantly dopaminergic (DA) neurons [7]. The VTA sends DA projections to both limbic and cortical areas and forms two important circuits: the mesolimbic circuit (VTA to the nucleus accumbens, VTA-to-NAc) and the mesocortical circuit (VTA to the medial prefrontal cortex, VTA-to-mPFC) [8, 9].

Both increases and decreases in the firing activity of VTA DA neurons have been reported in chronic pain states [10–12]. Theses discrepancies may be explained by the intricate heterogeneity of the different DA projection neurons. There is evidence that the firing activity of VTA DA neurons modulates the functional output of the reward system through its downstream targets [13, 14]. As such, the VTA-to-NAc and VTA-to-mPFC DA neurons may undergo differential adaptations in chronic pain states. Furthermore, the NAc and the mPFC, at the single-brain-region level, have been studied extensively for their roles in the processing of nociception under physiological and pathological pain conditions [11, 15–18]. Therefore, we speculated that the mesolimbic and mesocortical circuits play different roles in regulating nociceptive responses in chronic pain states.

The roles of the mesolimbic DA neurons in regulating nociception are strongly associated with their neuronal activity [19]. We previously reported that the increased firing activity of the VTA-to-NAc DA neurons mediates the thermal hyperalgesia induced by chronic constriction injury (CCI) of the sciatic nerve by releasing brain-derived neurotrophic factor (BDNF) into the NAc [19]. However, the mechanisms underlying the changes of DA neuronal activity remain largely unknown.

K^+^ voltage-gated channel subfamily Q (KCNQ) channels are widely expressed in neurons and have been implicated in pain [20–24]. Several studies have shown that KCNQ channels are involved in the modulation of hyperalgesia by regulating neuronal activity in peripheral nerve [20, 21]. A recent report demonstrated that overexpression of KCNQ channels normalizes the hyperactivity of VTA DA neurons [25]. These findings led us to hypothesize that that KCNQ channels of the VTA DA neurons might be involved in the regulation of nociception in chronic pain states.

Using electrophysiological, chemogenetics, and pharmacological approaches, we explored the roles of the VTA-to-NAc and VTA-to-mPFC DA neurons in an animal model of CCI-induced neuropathic pain and further investigated the underlying cellular mechanism.

## Materials and Methods

### Animals

Male C57BL/6J mice aged 7–8 weeks were provided by the Experimental Animal Center of Xuzhou Medical University and were acclimated to the housing facility for 1 week prior to experiments. These mice were group-housed (5 per cage) and maintained on a 12-h light/dark cycle with controlled humidity and temperature, and *ad libitum* access to food and water. All experiments were performed during the light cycle and all the behavioral tests were conducted by double-blind randomization in a silent room. All procedures followed the recommendations of the National Institutes of Health Guide for Care and Use of Laboratory Animals as well as the Committee for Research and Ethical Issues of the International Association for the Study of Pain.

### CCI Surgery

CCI surgery was performed following a method described previously [26]. Briefly, mice were anesthetized with 1% pentobarbital sodium (40 mg/kg, i.p.) and the skin on the lateral surface of the left thigh was incised. The left sciatic nerve was exposed at mid-thigh level and a unilateral constriction injury just proximal to the trifurcation was performed with three loose ligatures using 4–0 silk (spaced at 1-mm intervals). In sham-operated animals, the nerve was exposed but not ligated. The incision was closed in layers, and the wound was treated with topical penicillin.

### Stereotaxic Injections

Mice were anesthetized with 1% pentobarbital sodium (40 mg/kg, i.p.) and then placed in a stereotaxic apparatus (RWD, China). The cranium was exposed with the periosteum removed by 3% hydrogen peroxide. To label the projection-specific VTA DA neurons, lumafluor (0.5 μL, Lumafluor Inc., Durham, NC) was injected either into the NAc (in mm): anterior/posterior (AP), +1.40; medial/lateral (ML), +0.60; dorsal/ventral (DV), −4.70 or into the mPFC (in mm): AP, +1.95; ML, +0.27; DV, −2.00. Retigabine (20 µmol/L, MB1200, Melone Pharmaceutical Co., Ltd., China) was injected into the VTA (in mm): AP, −3.30; ML, +1.05; DV, −4.60; 7º, in a volume of 0.3 μL to investigate the effect of retigabine on behavior associated with neuropathic pain. The viruses were from BrainVTA (Wuhan, China). To evaluate the expression of KCNQ2 and KCNQ3 in VTA-to-NAc and VTA-to-mPFC DA neurons, rAAV-TH-NLS-Cre-WPRE-pA (0.2 μL, 2/R, 5.65 × 10^12^ genomic copies per mL) and rAAV-Eflα-DIO-EGFP-WPRE-pA (0.2 μL, 2/9, 6.86 × 10^12^ genomic copies per mL) were respectively injected into the NAc or mPFC and the VTA. To modulate projection-specific VTA DA neurons by chemogenetics, rAAV-TH-NLS-Cre-WPRE-pA (0.2 μL, 2/R, 5.65 × 10^12^ genomic copies per mL) was injected into the NAc or mPFC, while rAAV-Eflα-DIO-hM4D(Gi)-mCherry-WPRE-pA (0.2 μL, 2/9, 5.67 × 10^12^ genomic copies per mL) or rAAV-Eflα-DIO-hM3D(Gq)-mCherry-WPRE-pA (0.2 μL, 2/9, 5.65 × 10^12^ genomic copies per mL) was injected into the VTA contralateral to the CCI surgery. To overexpress KCNQ2 in projection-specific DA neurons, rAAV-TH-NLS-Cre-WPRE-pA (0.2 μL, 2/R, 5.65 × 10^12^ genomic copies per mL) was injected into the NAc or mPFC, and rAAV-CMV-DIO-KCNQ2-EGFP (0.2 μL, 2/R, 5.65 × 10^12^ genomic copies per mL) into the VTA. To elucidate the role of BDNF signaling in retigabine-induced behavioral outcomes in the mesocorticolimbic system, 0.2 μL BDNF (10 ng, B3795; Sigma-Aldrich) was injected into the NAc or mPFC. All the reagents were microinjected using a Hamilton syringe needle (33-gauge) at 0.1 µL/min with a pump (Harvard Apparatus).

### Measurement of Thermal Nociception

The Hargreaves test [27] was performed to evaluate the thermal nociceptive response by using an IITC plantar analgesia meter (IITC Life Science, Inc.). In brief, mice were placed in plastic cages on a glass platform and allowed to acclimatize for 30 min before testing. A radiant heat source was positioned directly beneath the glass and focused on the plantar surface of the left hind paw. The nociceptive endpoint in the radiant heat test was the lifting or licking of the hind paw. The time from onset of the radiant heat to the reaction was considered as the paw withdrawal latency (PWL). The radiant heat intensity was adjusted at the beginning of the experiment to obtain a basal PWL of 12–15 s and the cutoff time for exposure was set at 25 s to prevent tissue damage. The measurements were repeated five times at 5-min intervals and the average of the five measurements was calculated. For the chemogenetic-mediated behavioral test, the animals were injected intraperitoneally with clozapine-N-oxide (CNO; 1 mg/kg, dissolved in saline; HY-17366, MCE) 30 min prior to the test.

### *Ex-vivo* Electrophysiological Recordings

All recordings were carried out blind to the experimental conditions of behavioral, drug, and viral treatments. Mice were anesthetized with isofluorane and perfused immediately with ice-cold aCSF (artificial cerebrospinal fluid) for 40–60 s, which contained (in mmol/L): 128 NaCl, 3 KCl, 1.25 NaH_2_PO_4_, 10 D-glucose, 24 NaHCO_3_, 2 CaCl_2_, and 2 MgCl_2_ (oxygenated with 95% O_2_ and 5% CO_2_, pH 7.4, 295–305 mOsm). Acute brain slices containing VTA DA neurons were cut on a microslicer (DTK-1000, Ted Pella) in ice-cold sucrose aCSF, in which the NaCl was fully replaced with 254 mmol/L sucrose and saturated by 95% O_2_ and 5% CO_2_. Slices were maintained in holding chambers with aCSF for 1 h recovery at 37°C. For experiments, slices were transferred to the recording chamber and perfused (2–2.5 mL/min) with oxygenated aCSF at 35°C.

Patch pipettes (3–5 MΩ) for whole-cell current-clamp, voltage-clamp, and cell-attached recordings were filled with internal solution containing the following (in mmol/L): 115 potassium gluconate, 20 KCl, 1.5 MgCl_2_, 10 phosphocreatine, 10 HEPES, 2 magnesium ATP, and 0.5 GTP (pH 7.2, 285 mOsm). VTA DA neurons were identified by their location and infrared differential interference contrast microscopy and recordings were made from lumafluor-positive neurons for projection-specific recordings.

Cell-attached recordings were used for measurements of the spontaneous activity of VTA DA neurons. Signals were band-pass filtered at 300 Hz–1 kHz to identify DA neurons and were then Bessel filtered at 10 kHz (gain 50) using a Multiclamp 700B amplifier (Molecular Devices). For chemogenetics validations, the spontaneous activity of VTA DA neurons was recorded continuously before, during, and after CNO perfusion (5 mmol/L).

To measure M-current, whole-cell recordings were carried out in voltage-clamp mode. The neurons were recorded in the presence of 1 μmol/L tetrodotoxin (TTX; Alomone Labs), at a holding potential of −20 mV, and 1-s repolarizing steps were then applied to −40 mV. To measure the intrinsic membrane properties of VTA DA neurons, whole-cell recordings were carried out in current-clamp mode and spikes were induced by 100-pA current injection.

Retigabine (10 µmol/L, MB1200, Melone Pharmaceutical Co., Ltd., China) was used to investigate the role of KCNQ channels in regulating the activity of VTA DA neurons. Series resistance was monitored during all recordings. Data were acquired and analyzed using a Digidata 1440 A digitizer and pClamp 10.2 (Molecular Devices).

### Immunohistochemistry

Under general anesthesia with 1% pentobarbital sodium (40 mg/Kg, i.p.), the mice were intracardially perfused with 30 mL cold PBS (pH 7.4) and 30 mL 4% paraformaldehyde. The brains were removed and post-fixed at 4°C overnight, then kept in 30% sucrose for 2 days. Coronal sections (30 µm) were cut on a freezing microtome (VT1000S, Leica Microsystems). VTA sections were directly imaged for lumafluor, EGFP, and mCherry expression on a laser scanning confocal microscope (FluoView FV1000; Olympus).

For immunofluorescence staining, the sections were blocked for 1 h with 3% donkey serum and 0.25% Triton X-100 in PBS at room temperature. Then they were incubated with the primary antibodies rabbit anti-Kv7.2 (KCNQ2, 1:100; APC-050, Alomone Labs), rabbit anti-Kv7.3 (KCNQ3, 1:100; APC-051, Alomone Labs), and mouse anti-tyrosine hydroxylase (1:600, 318; Millipore), at 4°C overnight. The sections were then incubated for 2 h at room temperature with the secondary antibodies Alexa Fluor 488 anti-rabbit (A-11034), AlexaFluor 594 anti-mouse (A-11032), and AlexaFluor 488 anti-mouse (A-32723) (1:200, Molecular Probes), and rinsed three times with PBS before mounting. Afterwards, the sections were visualized under an Olympus confocal microscope.

### Western Blot

NAc and mPFC tissue blocks were harvested with 12-gauge punches and then sonicated in 30 μL homogenization buffer containing 320 mmol/L sucrose, 5 nmol/L HEPES, 1% sodium dodecyl sulfate (v/v), phosphatase inhibitor cocktails I and II (Sigma-Aldrich), and protease inhibitors (Roche Diagnostics). Protein concentrations were determined with a DC protein assay (Bio-Rad). Proteins (20–40 μg protein per lane) were electrophoresed in 10% SDS-PAGE gel and transferred onto PVDF membranes. The membranes were incubated at 4°C overnight with the primary rabbit anti-BDNF (1:1000, sc-546, Santa Cruz) and mouse anti-GAPDH (1:1000, E021010, Earthox) antibodies. The membranes were rinsed and then incubated for 2 h at room temperature with the secondary antibodies conjugated to alkaline phosphatase (1:500; Santa Cruz). The immune complexes were detected with a BCIP/NBT kit (Beyotime). Blots were analyzed with Photoshop software (Adobe Systems, Inc.), and the gray-scale values of protein bands were normalized to those of GAPDH.

### Statistical Analysis

Data are presented as mean ± SEM. Statistical analyses were performed with GraphPad Prism 6.0 software (Graph Pad Software, Inc.). Differences among three or more groups were compared with one-way ANOVA followed by Tukey’s *post hoc* test. The unpaired *t* test was used if only two groups were compared. The significance of any difference in thermal PWLs in the behavioral tests was assessed with a two-way ANOVA with repeated measures and Bonferroni’s *post hoc* test. All *P* values given are based on two-tailed tests. For all analyses, *P* <0.05 was considered to be statistically significant.

## Results

### Changes of Firing Rates in Mesocorticolimbic DA Neurons Under Chronic Pain

**Fig. 1:**
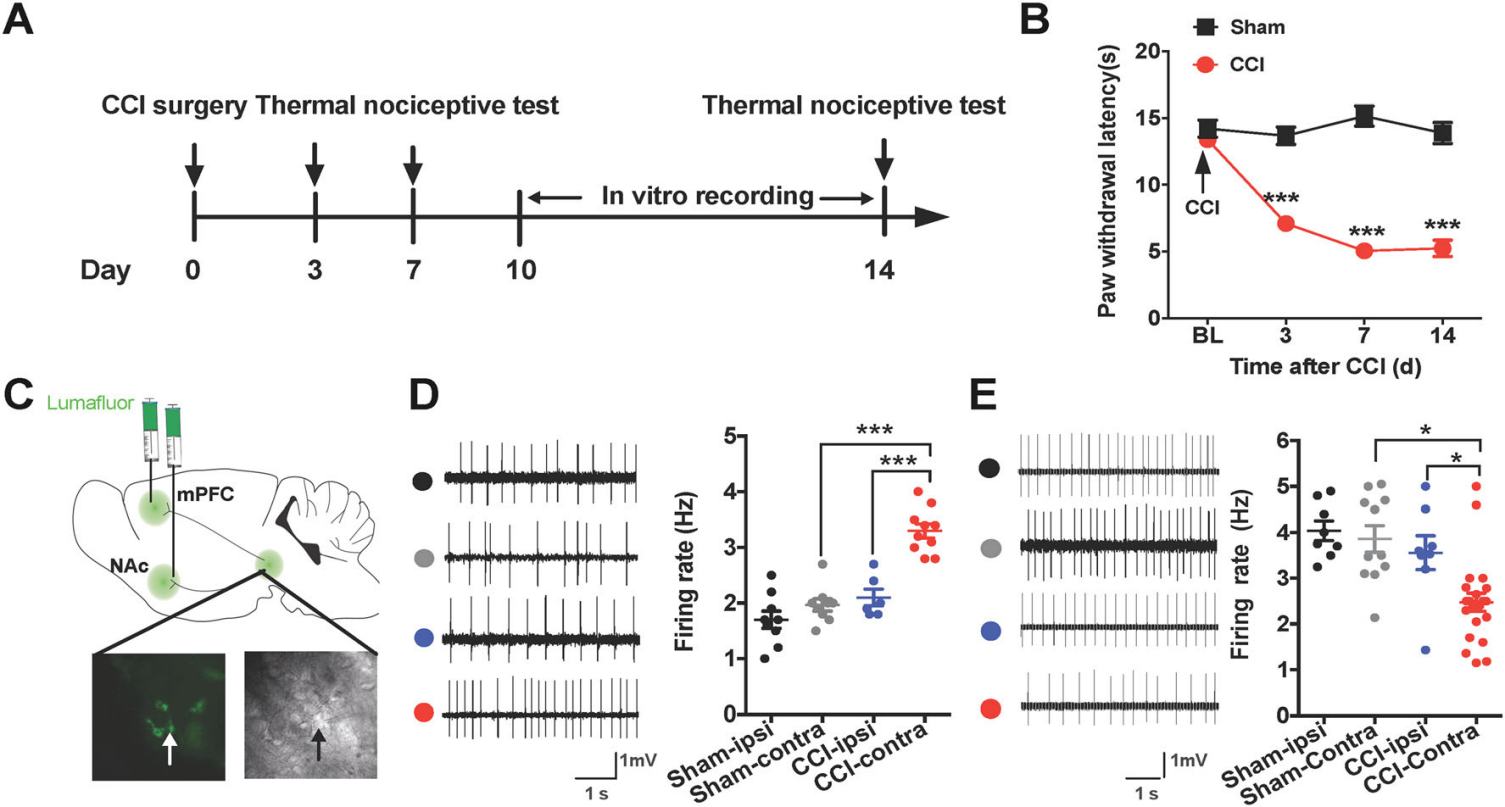
CCI induces different changes in the firing rates of VTA-to-NAc and VTA-to-mPFC DA neurons. **A** Experimental timeline. **B** Summary results showing that PWLs are lower on day 3, 7, and 14 after CCI surgery than in sham mice (*n* = 7 mice/group; ***P <0.001, two-way ANOVA with repeated measures and Bonferroni’s post-test). **C** Schematic of retrograde lumafluor injection into the NAc or mPFC and labeling lumafluor in the VTA (scale bar, 50 μm). **D** Sample traces and statistics of cell-attached recordings showing that CCI induces an increase in the firing rates of contralateral VTA-to-NAc DA neurons (*n* = 9–12 cells/3–4 mice/group; ***P <0.001 *vs* sham-contra and CCI-ipsi groups, one-way ANOVA with Tukey’s post-test). **E** Sample traces and statistics of cell-attached recordings showing that CCI induces a decrease in the firing rates of contralateral VTA-to-mPFC DA neurons (*n* = 10–22 cells/3–5 mice/group; *P <0.05 *vs* sham-contra and CCI-ipsi groups, one-way ANOVA with Tukey’s post-test). Error bars show mean and SEM.

PWLs were used to assess nociceptive responses [27]. CCI mice exhibited a significant decrease in the PWLs of the affected hind paws, which lasted for at least 14 days (Fig. 1A, B). To investigate the firing activity of the VTA-to-NAc and VTA-to-mPFC DA neurons in a state of CCI-induced chronic neuropathic pain, we injected the retrograde tracer lumafluor into the NAc or mPFC 7 days before CCI surgery to label projection-specific VTA neurons. Cell-attached electrophysiological recordings were performed in these labeled neurons, and the DA neurons were identified by their waveforms under signal-filtered condition (see Methods) (Fig. 1C). The *ex-vivo* recordings showed that CCI induced a significant increase in the firing activity in the contralateral, but not the ipsilateral, VTA-to-NAc DA neurons (Fig. 1D). In contrast, the firing activity of contralateral VTA-to-mPFC DA neurons was dramatically decreased (Fig. 1E). These data indicate that the VTA-to-NAc and VTA-to-mPFC DA neurons respond differentially to CCI-induced neuropathic pain.

### Roles of the Mesocorticolimbic Circuits in Regulation of CCI-Induced Thermal Nociception

**Fig. 2:**
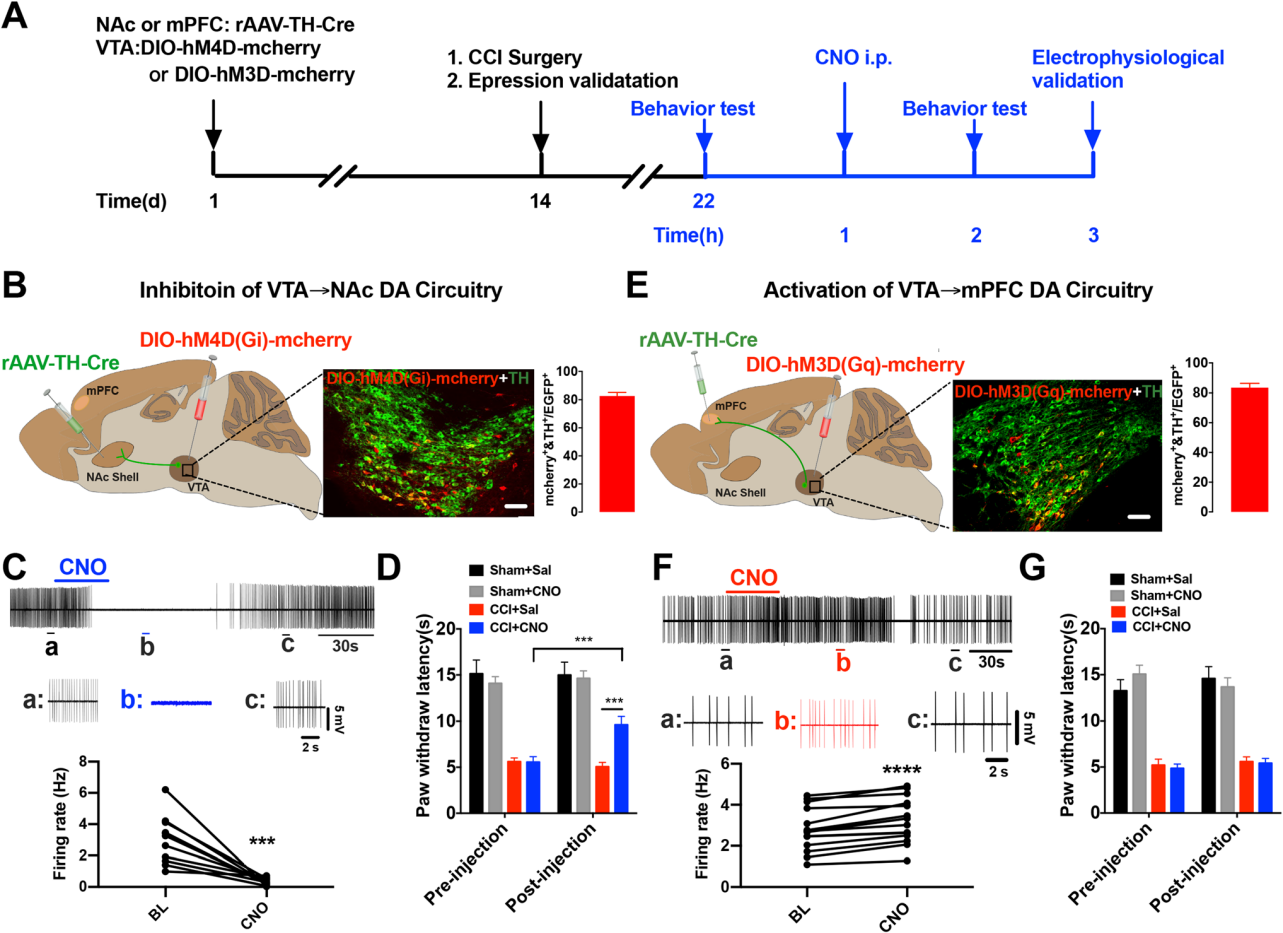
The VTA-to-NAc, but not the VTA-to-mPFC DA circuit, regulates thermal nociception in CCI mice. **A** Experimental timeline. **B** Schematic of viral injections of retrograde rAAV-TH-NLS-Cre-WAPE-pA into the NAc and cre-dependent rAAV-Eflα-DIO-hM4D(Gi)-mCherry-WPRE-pA into the VTA; representative confocal image showing co-expression of hM4Di expression (red) in VTA DA neurons (green) (scale bar, 200 μm). **C** Sample traces and statistics from VTA slices showing that the firing activity of putative VTA-to-NAc DA neurons are inhibited by CNO perfusion (*n* = 11 cells from 6 mice; ***P <0.001, paired *t* test). **D** Summary data showing that CCI mice with CNO injection exhibit alleviated nociceptive responses compared to mice with saline injection (n = 8–9 mice/group; ***P <0.001 *vs* pre-CNO injection and CCI+Sal groups, two-way ANOVA with repeated measures and Bonferroni’s post-test). **E** Schematic of viral injections of a retrograde rAAV-TH-NLS-Cre-WAPE-pA into the mPFC and cre-dependent rAAV-Eflα-DIO-hM3D(Gq)-mCherry-WPRE-pA into the VTA; representative confocal image showing co-expression of hM3Dq expression (red) in VTA DA neurons (green) (scale bar, 200 μm). **F** Sample traces and statistics from VTA slices showing that the firing of putative VTA-to-mPFC DA neurons is increased by CNO perfusion (*n* = 16 cells from 6 mice; ****P <0.0001, Wilcoxon test). **G** Summary data showing that CNO injection does not change the PWLs of CCI mice (*n* = 6–8 mice/group; two-way ANOVA with repeated measures and Bonferroni’s post-test). Error bars show the mean and SEM.

To answer the question whether these activity changes of the mesocorticolimbic DA neurons are implicated in regulation of thermal nociception in CCI-induced neuropathic pain, we first chemogenetically decreased the activity of VTA-to-NAc DA neurons by injection of a retrograde AAV, rAAV-TH-NLS-Cre-WAPE-pA, into the NAc, and a cre-dependent AAV, rAAV-Eflα-DIO-hM4D(Gi)-mCherry-WPRE-pA, into the VTA (Fig. 2A, B). The expression of hM4Di in VTA DA neurons was confirmed by co-localization of TH and mCherry (Fig. 2B) and its function was validated as a decreased firing rate of mCherry-labeled VTA DA neurons in acute slices induced by CNO perfusion (5 mmol/L) (Fig. 2C). CCI surgery was conducted 2 weeks following the microinjections, and 7 days after that, PWLs were measured. CCI mice with CNO injection (1 mg/kg, i.p.) showed increased PWLs (Fig. 2D), suggesting that the chemogenetic inhibition of VTA-to-NAc DA neurons reversed CCI-induced thermal hyperalgesia.

Next, we tested whether the decreased firing activity of the VTA-to-mPFC DA neurons contributed to the thermal nociception in CCI mice. To upregulate the firing activity in these neurons, we injected rAAV-TH-NLS-Cre-WAPE-pA into the mPFC, and rAAV-Eflα-DIO-hM3D(Gq)-mCherry-WPRE-pA into the VTA (Fig. 2A, E). The viral expression and its function were confirmed by immunofluorescence staining and cell-attached recordings (Fig. 2E, F). Unlike inhibition of the VTA-to-NAc DA neurons, this chemogenetic activation by CNO injection did not change the PWLs in CCI mice (Fig. 2G). These results suggest an involvement of the VTA-to-NAc, but not the VTA-to-mPFC DA neurons, in regulating thermal nociception under a state of chronic neuropathic pain.

### Changes of M-current (***I***_M_) in Mesocorticolimbic DA Neurons Under Chronic Pain

**Fig. 3:**
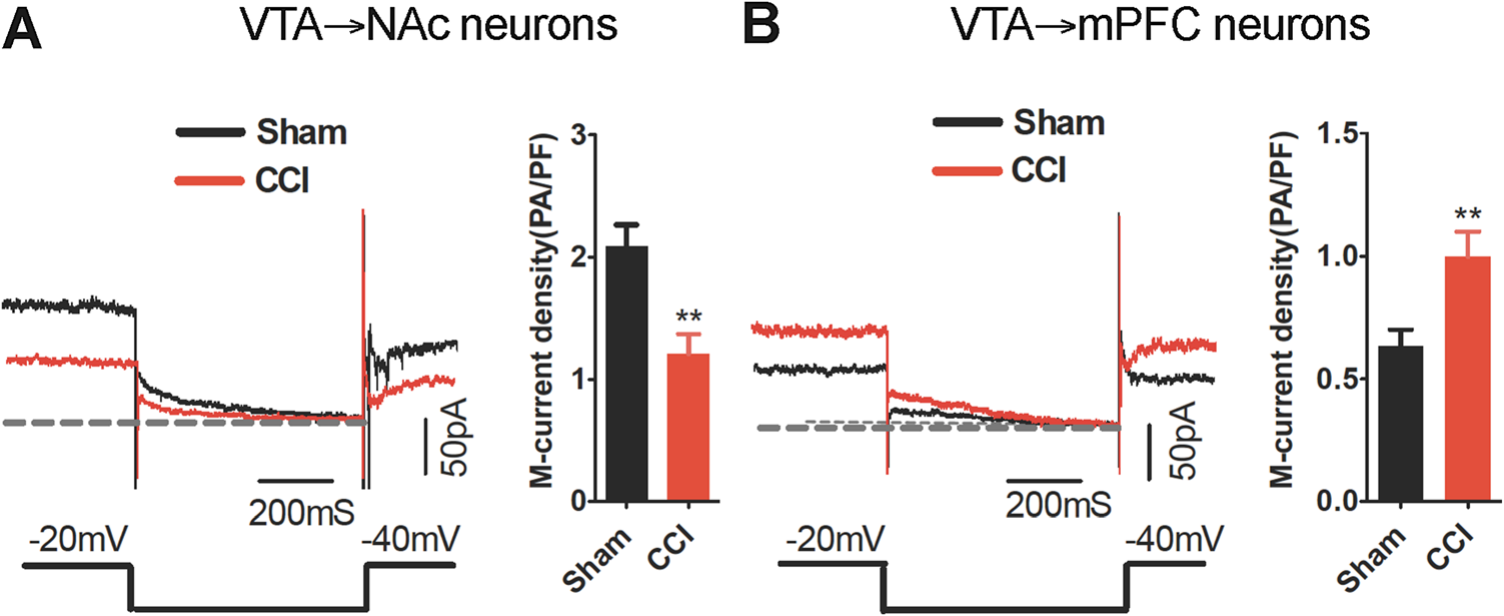
CCI induces different changes in the *I*_*M*_ of VTA-to-NAc and VTA-to-mPFC DA neurons. **A** Sample traces and statistics from VTA-to-NAc DA neurons showing decreased M-current density in CCI mice (*n* = 11 cells/3–4 mice/group; **P <0.01, unpaired *t* test). **B** Sample traces and statistics from VTA-to-mPFC DA neurons showing increased M-currents in CCI mice (*n* = 11–15 cells/3–5 mice/group; **P <0.01 *vs* sham group, unpaired *t* test).

*I*_M_, a voltage-gated K^+^ current, is involved in the physiological and pathological modulation of neuronal activity [28–30]. It is mediated by KCNQ-type K^+^ channels [28, 31] and thus measurement of *I*_M_ reflects the function of KCNQ channels. We used the retrograde tracer lumafluor to label VTA-to-NAc and VTA-to-mPFC neurons and performed *ex-vivo* whole-cell recordings to measure *I*_M_ in these labeled cells from CCI mice. The results showed that CCI mice exhibited decreased M-current density in the contralateral VTA-to-NAc DA neurons (Fig. 3A) and increased density the contralateral VTA-to-mPFC DA neurons (Fig. 3B), suggesting that CCI differentially modulates the KCNQ channels of mesocorticolimbic DA neurons, and thus may lead to the opposite changes of firing activity in the VTA-to-NAc and VTA-to-mPFC DA neurons.

### Location of KCNQ2/3 Channels in Mesocorticolimbic DA Neurons

**Fig. 4:**
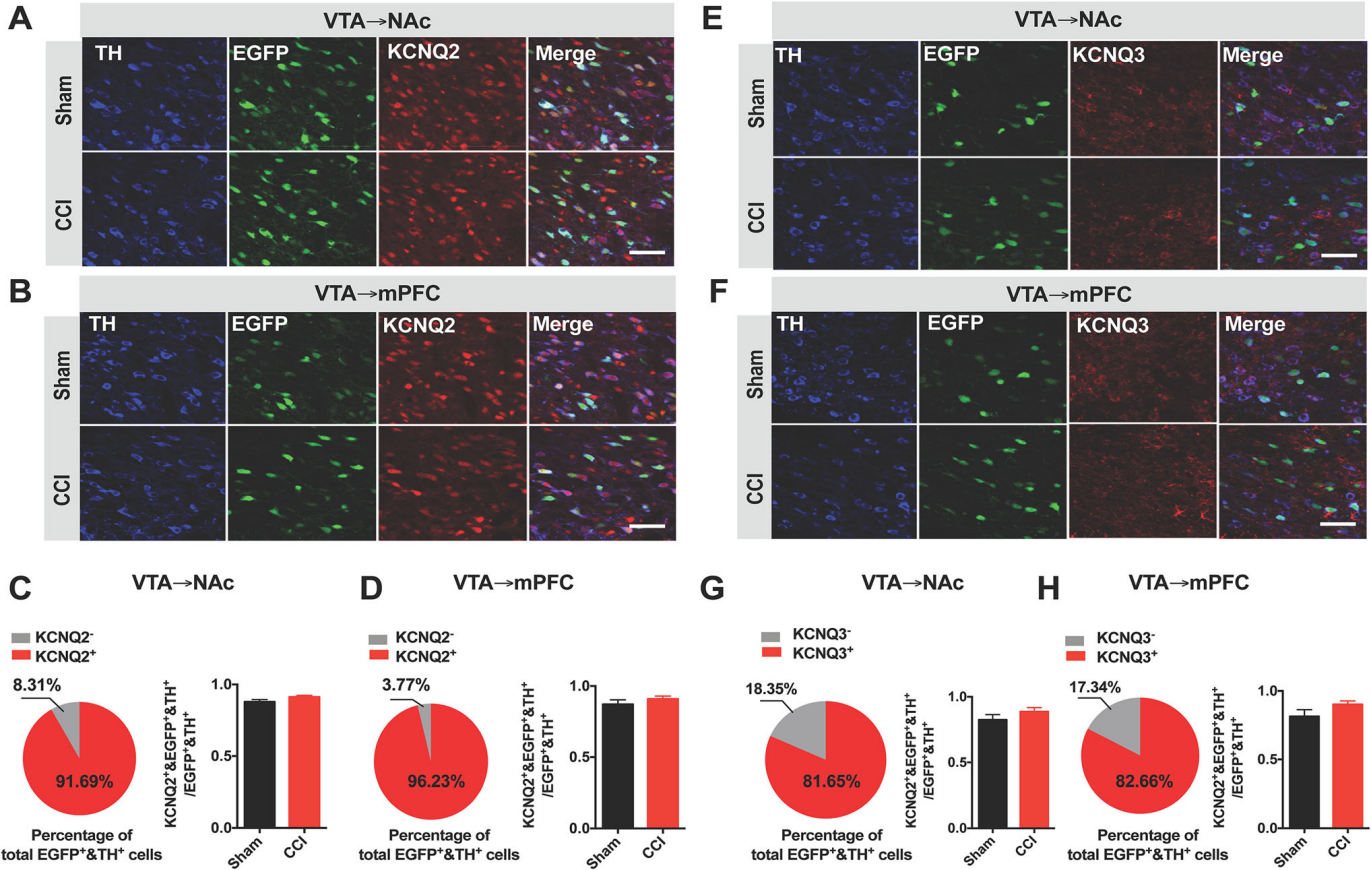
Location of KCNQ2 and KCNQ3 in VTA-to-NAc and VTA-to-mPFC DA neurons. **A** Confocal images showing the expression of KCNQ2 (red) in putative VTA-to-NAc DA neurons (blue and green) (scale bar, 50 μm). **B** Confocal images showing the expression of KCNQ2 (red) in the putative VTA-to-mPFC DA neurons (blue and green) (scale bar, 50 μm). **C** Statistics for the percentage of KCNQ2-positive cells in putative VTA-to-NAc DA neurons; the sham and CCI mice did not differ (2–3 sections/mouse from 3 mice; unpaired *t* test). **D** Statistics for the percentage of KCNQ2-positive cells in putative VTA-to-mPFC DA neurons; the sham and CCI mice did not differ (2–3 sections/mouse from 3 mice; unpaired *t* test). **E** Confocal images showing the expression of KCNQ3 (red) in putative VTA-to-NAc DA neurons (blue and green) (scale bar, 50 μm). **F** Confocal images showing the expression of KCNQ3 (red) in putative VTA-to-mPFC DA neurons (blue and green) (scale bar, 50 μm). **G** Statistics for the percentage of KCNQ3-positive cells in putative VTA-to-NAc DA neurons; the sham and CCI mice did not differ (2–3 sections/mouse from 3 mice; unpaired *t* test). **H** Statistics for the percentage of KCNQ3-positive cells in putative VTA-to-mPFC DA neurons; the sham and CCI mice did not differ (2–3 sections/mouse from 3 mice; unpaired *t* test). Error bars show the mean and SEM.

Immunohistochemical studies have shown that KCNQ2 and KCNQ3 channels are present in VTA neurons [30, 32–34]. Thus, we investigated the location of KCNQ2/3 channels in mesocorticolimbic DA neurons. To do this, we labeled the two DA populations by microinjection of a retrograde AAV, rAAV-TH-NLS-Cre-WAPE-pA, into the NAc or mPFC, and a cre-dependent AAV, rAAV-Eflα-DIO-EGFP-WPRE-pA, into the VTA. The EGFP- and TH-positive cells were concerned as the putative circuit-specific DA populations. KCNQ2-labeled cells constituted 91.69% of VTA-to-NAc DA neurons (Fig. 4A, C), and 96.23% of VTA-to-mPFC neurons (Fig. 4B, D). Meanwhile, 81.65% of the VTA-to-NAc DA neurons (Fig. 4E, G) and 82.66% of the VTA-to-mPFC population (Fig. 4F, H) were KCNQ3-positive.

We further analyzed the proportions of KCNQ2- or KCNQ3-positive cells in VTA-to-NAc or VTA-to-mPFC DA neurons. No significant differences were seen between the CCI mice and the sham counterparts (Fig. 4A–H). These data suggest no quantitative changes of the DA neurons that contain KCNQ2/KCNQ3 channels in CCI-induced chronic pain. It is worth noting that this result does not deny differential expression of KCNQ2/KCNQ3 channels in the two VTA DA projections in chronic neuropathic pain.

### Roles of Circuit-Specific KCNQ2 Overexpression in Regulating Thermal Nociception

**Fig. 5:**
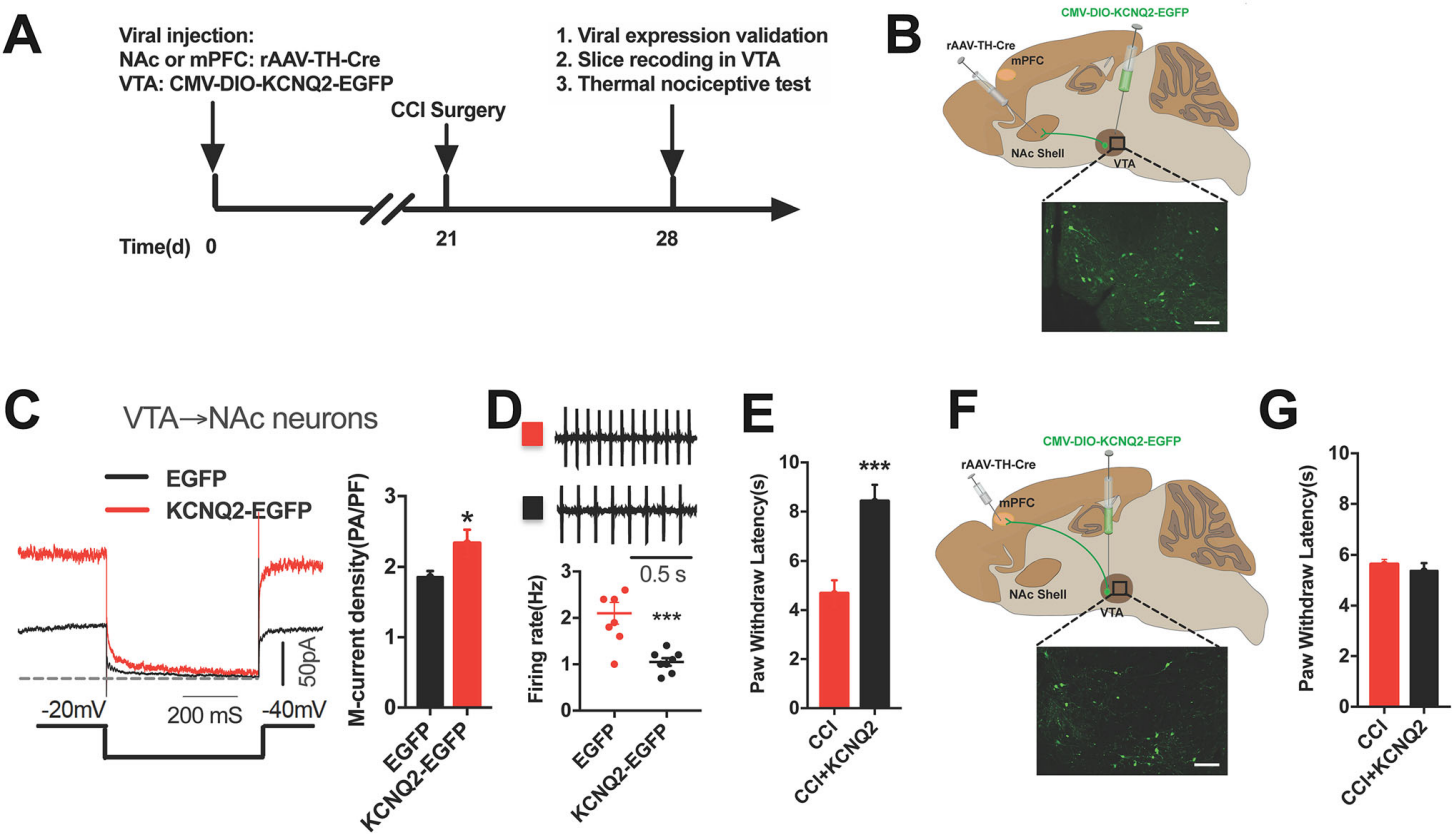
KCNQ2 overexpression in the VTA-to-NAc, but not the VTA-to-mPFC DA neurons relieves thermal nociception in CCI mice. **A** Experimental timeline. **B** Schematic of viral injections of a retrograde rAAV-TH-NLS-Cre-WAPE-pA into the NAc, and a cre-dependent rAAV-CMV-DIO-KCNQ2-EGFP-WPRE-pA into the VTA; representative confocal image showing KCNQ2-EGFP expression (green) in the VTA (scale bar, 100 μm). **C** Sample traces and statistics from VTA-to-NAc DA neurons showing increased M-current density in CCI mice with KCNQ2 overexpression in VTA-to-NAc DA neurons (*n* = 10–22 cells/3–5 mice/group; *P <0.05, unpaired *t* test). **D** Sample traces and summary showing that the firing rate of VTA-to-NAc DA neurons was decreased in CCI mice with KCNQ2 overexpression in these neurons (*n* = 10–22 cells/3–5 mice/group; ***P <0.001, unpaired *t* test). **E** Summary showing PWLs are increased after KCNQ2 overexpression in VTA-to-NAc DA neurons (*n* = 8 mice/group; ***P <0.001, unpaired *t* test). **F** Schematic of viral injections of retrograde rAAV-TH-NLS-Cre-WAPE-pA into the mPFC, and cre-dependent rAAV-CMV-DIO-KCNQ2-EGFP-WPRE-pA into the VTA; representative confocal image showing KCNQ2-EGFP expression (green) in the VTA (scale bar, 100 μm). **G** Summary data showing that KCNQ2 overexpression in the VTA-to-mPFC DA neurons does not change the PWLs in CCI mice (*n* = 8 mice/group; unpaired *t* test). Error bars show the mean and SEM.

Changes in both KCNQ expression and channel status may contribute to *I*_M_ alterations. To explore the roles of KCNQ channels in regulating thermal nociception in a chronic pain state, we overexpressed KCNQ2 in each of the two specific DA populations by injecting rAAV-TH-cre into the NAc or mPFC, and rAAV-CMV-DIO-KCNQ2-EGFP into the VTA (Fig. 5A, B, F). After validation of viral expression (Fig. 5B, F), whole-cell recordings confirmed that this projection-selective overexpression of KCNQ2 increased the M-current density (Fig. 5C) and decreased the firing activity (Fig. 5D). Behavioral results showed that KCNQ2 overexpression in the VTA-to-NAc DA neurons reversed the established thermal hyperalgesia in CCI mice as evidenced by the increased PWLs (Fig. 5E). This reversal was not seen in the mice with KCNQ2 overexpression in the VTA-to-mPFC DA neurons (Fig. 5G), These results indicate that functional modulation of KCNQ2 channels in the VTA-to-NAc, rather than the VTA-to-mPFC circuit contributes to regulating thermal nociception in chronic neuropathic pain.

### Relief of CCI-Induced Thermal Nociception by Retigabine

**Fig. 6:**
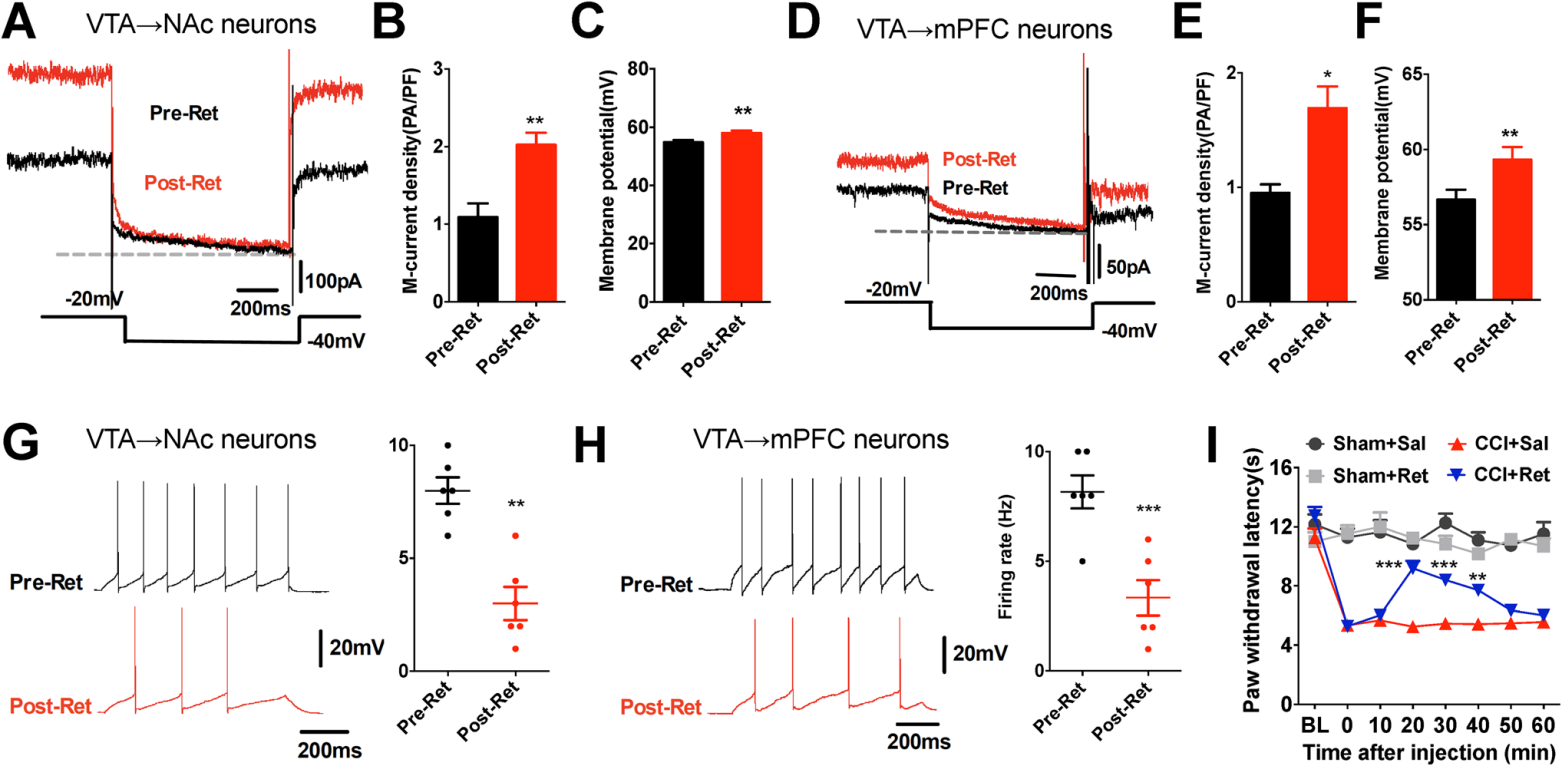
Intra-VTA injection of retigabine reduces nociceptive responses in CCI mice. **A**–**C** Sample traces **(A)** and quantitative data **(B**, **C)** showing that retigabine increases the M-current density and membrane potential in VTA-to-NAc DA neurons (*n* = 12–16 cells/3–4 mice/group; **P <0.01, unpaired *t* test). **D**–**F** Sample traces **(D)** and quantitative data **(E**, **F)** showing that retigabine increase the M-current density and membrane potential in VTA-to-mPFC DA neurons (*n* = 12–16 cells/3–4 mice/group, *P <0.05, **P <0.01, unpaired *t* test). **G** Sample traces and statistics from whole-cell recordings (100 pA current injection) showing decreased activity of VTA-to-NAc DA neurons (*n* = 6 cells/3 mice/group; **P <0.01, unpaired *t* test). **H** Sample traces and statistics from whole-cell recordings (100 pA current injection) showing decreased activity of VTA-to-mPFC DA neurons (*n* = 6 cells/3 mice/group; **P <0.01, unpaired *t* test). **I** Summary data showing that intra-VTA injection of retigabine increases PWLs in CCI mice (*n* = 6 mice/group; ***P <0.001, **P <0.01 *vs* CCI+Sal group, two-way ANOVA with repeated measures and Bonferroni’s post-test). Error bars show the mean and SEM.

Given that the functional status of KCNQ channels may also affect *I*_M_, we further investigated their effects on thermal nociception by activating KCNQ channels. The KCNQ opener retigabine, an FDA-approved agent for anticonvulsant therapy [35–37], was used here. The results from *ex-vivo* whole-cell recordings in VTA slices showed that perfusion of retigabine significantly increased the M-current density and membrane potential (Fig. 6A–F), and decreased the firing rate (Fig. 6G, H) in both VTA-to-NAc and VTA-to-mPFC DA neurons as compared with the vehicle controls, suggesting its non-selective effects on the mesocorticolimbic circuits. Furthermore, intra-VTA injection of retigabine (10 μmol/L, 0.15 μL) produced a clear anti-nociceptive response as evidenced by the increased PWLs (Fig. 6I). Combined with our previous finding, we suggest that retigabine exerts its anti-nociceptive effects by decreasing the activity of the VTA-to-NAc DA neurons by activating KCNQ channels.

### Mesocorticolimbic BDNF Signaling in the Anti-nociceptive Effect of Retigabine

**Fig. 7:**
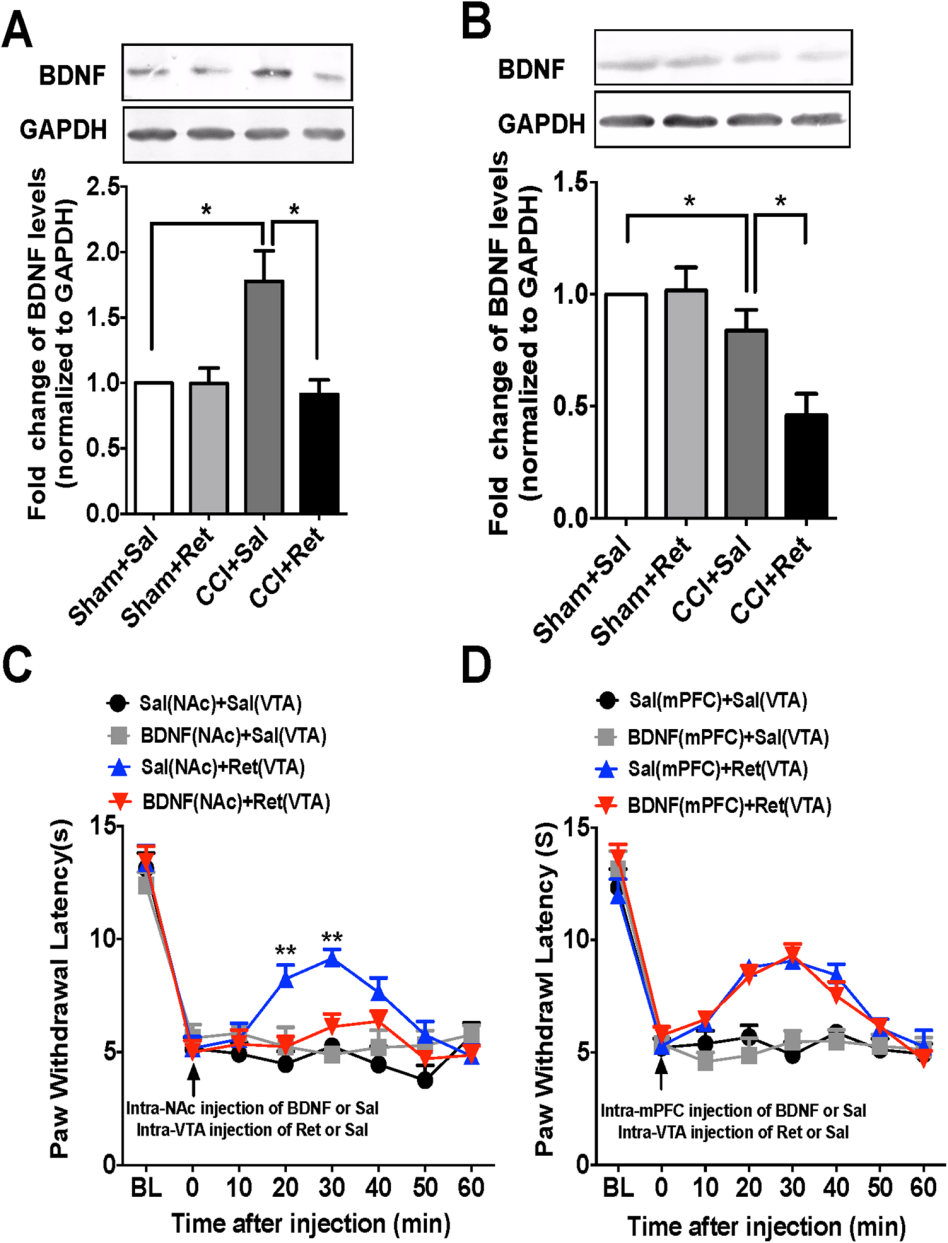
BDNF signaling in the VTA-to-NAc DA circuit is involved in the anti-nociceptive effect of retigabine. **A** Typical bands and summary showing upregulated BDNF protein levels in the NAc of CCI mice (*n* = 3; *P <0.05 *vs* sham+Sal group, one-way ANOVA with Tukey’s post-test); this was reversed by retigabine injection into the VTA (*n* = 3; *P <0.05 *vs* CCI+Sal group, one-way ANOVA with Tukey’s post-test). **B** Typical bands and summary showing downregulated BDNF protein levels in the mPFC of CCI mice (*n* = 3; *P <0.05 *vs* sham+Sal group, one-way ANOVA with Tukey’s post-test); this was further reduced by retigabine injection into the VTA (*n* = 3; *P <0.05 *vs* CCI+Sal group, one-way ANOVA with Tukey’s post-test). **C** Summary data showing that intra-NAc injection of exogenous BDNF abolishes the anti-nociceptive effect of intra-VTA retigabine injection in CCI mice (*n* = 6 mice/group; **P <0.01 *vs* Sal (NAc) + Ret (VTA) group, two-way ANOVA with repeated measures and Bonferroni’s post-test). **D** Summary data showing that intra-mPFC injection of exogenous BDNF does not alter the anti-nociceptive effect of retigabine in CCI mice (*n* = 6 mice/group; two-way ANOVA with repeated measures and Bonferroni’s post-test). Error bars show the mean and SEM.

BDNF plays important roles in regulating synaptic plasticity and neuronal survival [38, 39]. Previous studies have suggested that VTA DA neurons release BDNF into the targeted areas in an activity-dependent manner [38, 40]. Our recent work showed that BDNF in the VTA-to-NAc circuit is increased, thus mediating thermal nociception in mice with CCI-induced neuropathic pain [19]. Therefore, we asked whether BDNF signaling in the VTA-NAc circuit is involved in the anti-nociceptive effect of retigabine. Western blot assays showed that the BDNF protein levels in the NAc were drastically increased in CCI mice and this increase was reversed by intra-VTA injection of retigabine (Fig. 7A), suggesting that intra-VTA injection of retigabine decreased BDNF levels in the NAc under chronic pain. Furthermore, exogenous administration of BDNF into the NAc abolished the anti-nociceptive effect of intra-VTA retigabine injection in CCI mice (Fig. 7C). Unlike that in the NAc, CCI induced a significant decrease of BDNF protein levels in the mPFC (Fig. 7B). Intra-VTA injection of retigabine further lowered the BDNF levels in the mPFC in CCI mice (Fig. 7B). Exogenous BDNF injected into the mPFC did not alter the anti-nociceptive effect induced by intra-VTA retigabine injection (Fig. 7D). These data imply a potential role of BDNF signaling in the VTA-to-NAc DA circuit in the anti-nociceptive effect of retigabine on chronic neuropathic pain.

## Discussion

Our current results demonstrate that: (1) CCI differentially modulated the firing activity of the VTA-to-NAc and VTA-to-mPFC DA circuits, and of the two, the former is involved in thermal nociception regulation; (2) both KCNQ2 overexpression in the VTA-to-NAc DA neurons and intra-VTA injection of the KCNQ opener retigabine corrected mesolimbic KCNQ channel-mediated M-currents and DA neuronal activity, and relieved CCI-induced thermal nociception; and (3) BDNF signaling in the VTA-to-NAc DA circuit may be involved in the anti-nociceptive effect of retigabine. These findings reveal a novel KCNQ channel-based ionic and circuit mechanism for the regulatory roles of mesocorticolimbic circuits in chronic neuropathic pain-induced thermal nociception.

VTA DA neurons are a heterogeneous population divided into several subgroups in terms of their targets [13, 41]. Among them, the VTA-to-NAc and the VTA-to-mPFC DA neurons have been shown to have distinct properties: for example, these subpopulations have different firing properties [42, 43]. Specifically, the VTA-to-mPFC DA neurons discharge faster than the VTA-to-NAc DA neurons [42, 43], as confirmed by our current results (Fig. 1C–E).

Pain is a stressor, causing multiple adaptive changes in the brain [44, 45]. A growing body of literature suggests that the mesocorticolimbic DA circuits are implicated in chronic pain [3, 6, 19, 25, 46]. Similar stress can induce different firing alterations in these projection-specific VTA DA neurons [47]. For instance, recent studies have shown that the VTA-to-NAc and VTA-to-mPFC DA neurons exhibit distinct properties and actions in regulating stress-related depression [48–51]. Likewise, our current study reports such heterogeneity in CCI-induced chronic neuropathic pain. In line with our previous study [19], the current results suggest that the mesolimbic DA circuit is required for the regulation of thermal nociception in a state of chronic pain (Fig. 1, 2). Although the firing activity of VTA-to-mPFC DA neurons was decreased by CCI (Fig. 1E), activation of these neurons did not change the thermal nociceptive threshold (Fig. 2E–G). This result does not support an involvement of VTA-to-mPFC DA neurons in regulating the thermal nociception underlying chronic pain. It should be noted that the function of the decreased activity in the mesocortical DA neurons remains unknown. Based on recent studies [50, 52], we speculate that this adaptive change may be involved in regulating chronic pain-related negative emotions and cognitive impairments.

It has been shown that KCNQ channels serve as a brake to suppress abnormal ectopic discharges of neurons and control neuronal hyperexcitability [25, 28, 33]. These channels and related M-currents set the resting membrane potential [53], and exert an inhibitory effect on neuronal discharges in brain slice, spinal cord, and dorsal root ganglion neurons [20, 25, 54]. Structurally, KCNQ channels consist of five subunits as homo- or hetero-tetramers [28, 31, 33]. Among them, KCNQ2/3 channels are well recognized to form the molecular basis for M-currents [31], and have been found to affect DA neuronal activity within the VTA [32, 55]. It is known that the KCNQ2/3 heteromers exhibit an 11-fold larger current than the KCNQ2 or KCNQ3 homomers [56]. Based on their abundant distribution (Fig. 4), we speculate that the majority of KCNQ2 and KCNQ3 channels form heteromers in the VTA. Furthermore, both KCNQ2 overexpression and microinjection of the KCNQ opener retigabine corrected the CCI-induced changes of M-currents and firing activity in the VTA-to-NAc DA neurons and reversed thermal hyperalgesia. These data support the hypothesis that chronic neuropathic pain may heterogeneously modulate the expression and/or functional status of KCNQ channels in mesocorticolimbic DA circuits, resulting in the distinct firing properties. Although KCNQ4 and KCNQ5 may contribute to M-currents likewise, we did not test their roles due to their locations [57, 58].

The functional heterogeneity of the mesocorticolimbic DA circuits is largely dependent on changes of the firing activity in the VTA DA subpopulations [50, 51], and yet various ion channels expressed within these neurons play an important role as intrinsic factors in regulating firing activity [59]. Studies have shown that hyperpolarization-activated cation channel-mediated currents (*I*_h_) provide an excitatory driving force [60–62] and K^+^ channel-mediated currents provide an inhibitory driving force in VTA DA neurons [30, 63], and the balance between them is essential for normal function of the mesocorticolimbic DA circuits [55, 63, 64]. External stimuli may shift this balance towards an excitatory or inhibitory state in different projecting circuits [55], and then result in heterogeneous functional phenotypes. The ion channel-related heterogeneous alterations in mesocorticolimbic DA circuits have been well studied in the social defeat stress-induced depression model [25, 55]. We report similar ion channel-mediated functional heterogeneity of the mesocorticolimbic DA circuits in chronic pain processing. In detail, KCNQ channel-mediated M-type K^+^ currents, as an inhibitory driving force [30, 63], display heterogeneous alterations in the mesocorticolimbic DA circuits in mice with CCI-induced neuropathic pain (Fig. 3), which may result in changes of the firing activity of DA neurons.

Our previous study has shown that the VTA-to-NAc DA neurons exhibit an increase of the excitatory driving force *I*_h_ current in CCI mice and pharmacologically inhibiting VTA DA neuron firing with *I*_h_ blockers reverses CCI-induced NAc neuronal activation and thermal hyperalgesia in CCI mice [19]. Thus, we speculate that long-lasting nociceptive inputs with chronic pain shift the balance towards an excitatory state in the VTA-to-NAc DA circuit *via* modulating *I*_h_ and M-type K^+^ currents and therefore increase the firing activity of these neurons. These studies strongly support the conclusion that the intrinsic firing activity of VTA DA neurons serves as an important mechanism underlying the adaptations involved in chronic pain, and may provide valuable therapeutic targets for chronic pain.

Retigabine is an FDA-approved anticonvulsant drug for partial epilepsies. It reduces neuronal excitability in animals and humans by enhancing *I*_M_ *via* the activation of KCNQ channels [35]. Recent studies have also implicated this drug in treating lateral sclerosis [65], alcohol addiction [66], chronic pain [20–22], and depression [25]. Administration of retigabine has been reported to reverse mechanical and thermal hypersensitivity in inflammatory, neuropathic, and cancer pain and chemotherapy-induced peripheral neuropathy models [20–22, 24]. Retigabine exerts an analgesic effect through peripheral mechanisms including inhibiting the excitability of nociceptive neurons and C-type nerve fibers [20, 24]. Studies have also reported that intra-thalamic or intra-cerebral ventricular injection of retigabine produce an analgesic effect [67, 68], suggesting that a supraspinal mechanism may be involved in the anti-nociceptive effect of retigabine. In the present study, intra-VTA injection of retigabine alleviated CCI-induced thermal nociception, in parallel with increased M-currents and decreased firing activity in VTA DA neurons (Fig. 6). Combined with the finding that the adaptive changes in the VTA-to-NAc DA neurons contribute to CCI-induced thermal nociception, we speculate that correction of the KCNQ-mediated firing activity in VTA-to-NAc DA neurons may underlie the anti-nociceptive effect of retigabine.

Previous studies have demonstrated that BDNF is a critical nociceptive modulator in the mesolimbic reward circuit [19, 51]. There is a strong association between VTA DA neuronal firing rates and BDNF levels in the target regions [19, 38, 40]. We provide evidence that intra-VTA injection of retigabine corrected BDNF protein levels in the NAc in CCI mice (Fig. 7A, B). As BDNF levels in the NAc may reflect the VTA DA neuronal activity, this change in BDNF levels is in line with the finding that retigabine decreased the firing activity of the VTA DA neurons in CCI mice (Fig. 6G, H). In addition, exogenous BDNF in the NAc abolished the effect of retigabine on thermal nociception in CCI mice (Fig. 7C). These results reflect a possible role of BDNF signaling of the VTA-to-NAc DA circuit in the anti-nociceptive effect of retigabine. It is worth noting that retigabine similarly changed the BDNF levels in the mPFC, whereas exogenous BDNF delivered to the mPFC had no effect on thermal nociception (Fig. 7B, D). This point needs future investigations on the part played by the retigabine-induced decrease in BDNF levels in the mPFC.

Since pain is a distressing experience with emotional and cognitive components, a limitation of this study is that we only focused on thermal pain sensation. Whether and how this system is involved in processing pain-related negative emotions and cognition impairments remain to be explored. Limitations in viral expression may also occur due to nonspecific expression and the influence of injection sites.

In summary, our study reveals the functional heterogeneity of the mesocorticolimbic DA circuits in regulating thermal nociception in the chronic pain state, and unmasks a novel mechanism by which mesolimbic KCNQ channels regulate thermal nociception by modulating the VTA-to-NAc DA neuronal firing activity.
